# Transcatheter Embolization of Peripheral Renal Artery for Hemorrhagic Urological Emergencies using FuAiLe Medical Glue

**DOI:** 10.1038/srep09106

**Published:** 2015-03-13

**Authors:** Tianzhi An, Shasha Zhang, Min Xu, Shi Zhou, Weiping Wang

**Affiliations:** 1 Section of Interventional Radiology, Affiliated Hospital of Guiyang Medical College, 28 Guiyi Street, Guiyang, Guizhou, 550004 China; 2 Section of Radiology, Guizhou Provincial People's Hospital, 1 East Zhongshan Road, Guiyang, Guizhou, 550002 China; 3 Imaging Institute, Section of Interventional Radiology, Cleveland Clinic, 9500 Euclid Ave, Cleveland, OH. 44195

## Abstract

Our objective was to review the technical success and clinical outcomes of transcatheter embolization of peripheral renal artery with FuAiLe medical glue (FAL). All patients who underwent FAL embolization for peripheral renal artery bleeding were retrospectively analyzed for underlying pathologies, technical success and outcome of embolization procedure. 14 consecutive patients underwent FAL embolization between November 2009 and February 2013. The causes of bleeding were post biopsy (n = 5), blunt trauma (n = 5), percutaneous lithotripsy of kidney stones (n = 3), and complication of cardiac catheterization (n = 1). Bleeding was effectively controlled with a single injection of FAL. Mean volume of FAL mixture (FAL:Lipiodol, 1:1) was 0.5 mL (range, 0.2–0.8 mL). No reflux of the embolic agent was noted. Average cost of FAL for each procedure was $74. Postembolization clinical follow-up showed no evidence of recurrent hematuria, progression of hematoma, hypertension, or elevation of serum creatinine. Doppler ultrasound examinations in 13 patients demonstrated no abscess, renal parenchyma infarction, or renal artery abnormalities. Superselective FAL embolization may be used for the treatment of active bleeding from peripheral renal arteries. It has a high success rate and is quicker and less expensive than embolization with other agents.

Renal arterial injury can be caused by percutaneous renal biopsy, laparoscopic or robotic partial nephrectomy, percutaneous nephrostomy tube placement, percutaneous nephrostolithotomy, abdominal trauma, and open surgery^1^,^2^,^3^. Renal artery bleeding from these injuries can be significant and life-threatening, although most cases of renal artery bleeding are self-limiting and do not require intervention^4^. Immediate treatment is indicated in cases of massive bleeding, progression of retroperitoneal hematoma, or continuous hematuria with or without clinical symptoms. Control of renal arterial bleeding can be achieved through open surgical procedures such as partial or total nephrectomy or arterial ligation^5^. However, endovascular embolization is now considered the most appropriate technique because of its less invasive nature and high success rate^6^. Superselective renal embolization has been reported to be effective not only for the treatment of iatrogenic and penetrating vascular renal injuries but also for the preservation of the renal parenchyma^1^,^7^.

Coils are the embolic agents most commonly used to treat renovascular injuries. However, undesired embolic results can occur if an inappropriate coil size is selected, including difficult delivery and packing of the coil, coil migration, and vascular recanalization^8^,^9^. Glue is a liquid embolic agent that is not commonly used for embolization of the renal artery, primarily because the use of this agent is technically demanding and relatively expensive^10^,^11^,^12^,^13^. This study reports our experience of selective embolization of bleeding renal artery with FuAiLe medical glue (FAL) (Fuaile, Beijing, China) as a primary embolic agent.

## Methods

This retrospective study was approved by the institutional review board and the ethics committee of Affiliated Hospital of Guiyang Medical College. Informed consent was obtained from all patients before procedures. This study included all patients who underwent FAL embolization for renal arterial bleeding at our institution between November 2009 and February 2013. The methods were carried out in accordance with the approved guideline. All images were collected from our imaging archiving system, including abdominal computed tomography (CT) scans and arteriograms. Procedure logs were reviewed to obtain information about procedure time and cost of embolic agents. Postembolization Doppler ultrasound examinations were collected and analyzed. Clinical information was obtained from medical charts, clinic visits, and telephone interviews.

When acute renal arterial bleeding was suspected, patients underwent a CT scan with or without intravenous contrast depending on the physician's experience. Renal arterial embolization was considered based on the findings of this initial CT scan, laboratory results, and clinical signs of acute bleeding, such as decreased blood pressure and pulse. Selective renal arteriography was performed to assess the site and feeding pedicles, flow pattern, and venous drainage of the renal vascular lesion. Subselective branch arteriography was indicated if there was any suspicion of source of bleeding on the main renal arteriography. Superselective renal arterial embolization was carried out in patients with active contrast extravasation, renal arterial pseudoaneurysm, and/or arteriovenous fistula. Once the source of the bleeding was identified, a microcatheter was advanced to the target vessel through the existing 5-Fr Cobra or Yashiro catheter. FAL was mixed with Lipiodol (Guerbet, France) (which can prolong the polymerization time and improve radiopacity) at a ratio of 1:1. Before embolization, a simulated injection with contrast medium was performed to calculate the maximal injecting volume and speed of the glue. This was carried out by injecting contrast medium (<2 mL) under fluoroscopy through a microcatheter with the tip proximal to the target lesion. If the injected volume and speed of contrast filled the target artery without reflux into proximal branches, this test injection determined the volume and speed of actual FAL mixture administration (although an additional 0.3-mL volume should be considered due to the intraluminal volume of the microcatheter that would not be used^9^). Immediately before glue injection, the microcatheter was flushed with a 5% dextrose solution to avoid premature polymerization. The glue was injected under fluoroscopic guidance. When the injection was completed, the microcatheter was removed immediately to avoid adherence of the catheter tip to the vascular wall. The microcatheter was then flushed immediately with 5% dextrose so that it could be used for repeat glue injections. Postembolization renal arteriography was obtained by injecting the existing 5-Fr catheter to determine the embolic result. Repeat embolization with glue or other materials was indicated if the underlying vessel remained patent.

The procedure time was defined as the time from successful cannulation of the main renal artery to the last angiography. Technical success of the procedure was defined as complete occlusion of all renovascular bleeding with arterial embolization as documented by arterial angiography at the end of the procedure. Clinical success was defined as the cessation of gross hematuria within 3 days, absence of recurrent hematuria, absence of need for blood transfusion, absence of further decrease in hemoglobin by > 1.5 g/dL (15 g/L), and absence of need for repeat embolization or subsequent surgery^5^.

After the procedure, complete blood count (CBC) was tested daily for 3 to 7 days to monitor the stability of hemoglobin and hematocrit levels. All patients also had clinical follow-up for persistent hematuria, recurrent bleeding, fever (>38.5°C), back pain, decrease in renal function (serum creatinine levels > 130 μmol/L), arterial hypertension (defined as systolic arterial pressure > 160 mm Hg and diastolic pressure > 90 mm Hg), and any embolic procedure-related complications. Postprocedural Doppler ultrasound examination of the selected kidney was obtained for evaluation of any perirenal fluid collection, renal parenchyma infarction or abscess, and major renal artery patency.

## Results

A total of 14 consecutive patients (10 men; 4 women) underwent embolization with FAL injection as the primary embolic agent for hemorrhagic urological emergency caused by renal artery injuries. The average age of study patients was 32.9 years (range, 12–62 y). The underlying etiologies of bleeding were as follows: renal biopsy (*n* = 4), blunt trauma (*n* = 6), percutaneous lithotripsy of kidney stones (*n* = 3), and complication of coronary catheterization (*n* = 1). Clinical presentations, CT scan findings, preprocedure laboratory results, and postprocedure follow-up findings are shown in Table 1.

Among these 14 patients, arteriography demonstrated pseudoaneurysm in 10 cases and active contrast extravasation in the remaining 4 cases. The bleeding locations were interlobar arteries (*n* = 9) and arcuate arteries (*n* = 5); all cases involved a solitary lesion.

**Figure 1:**
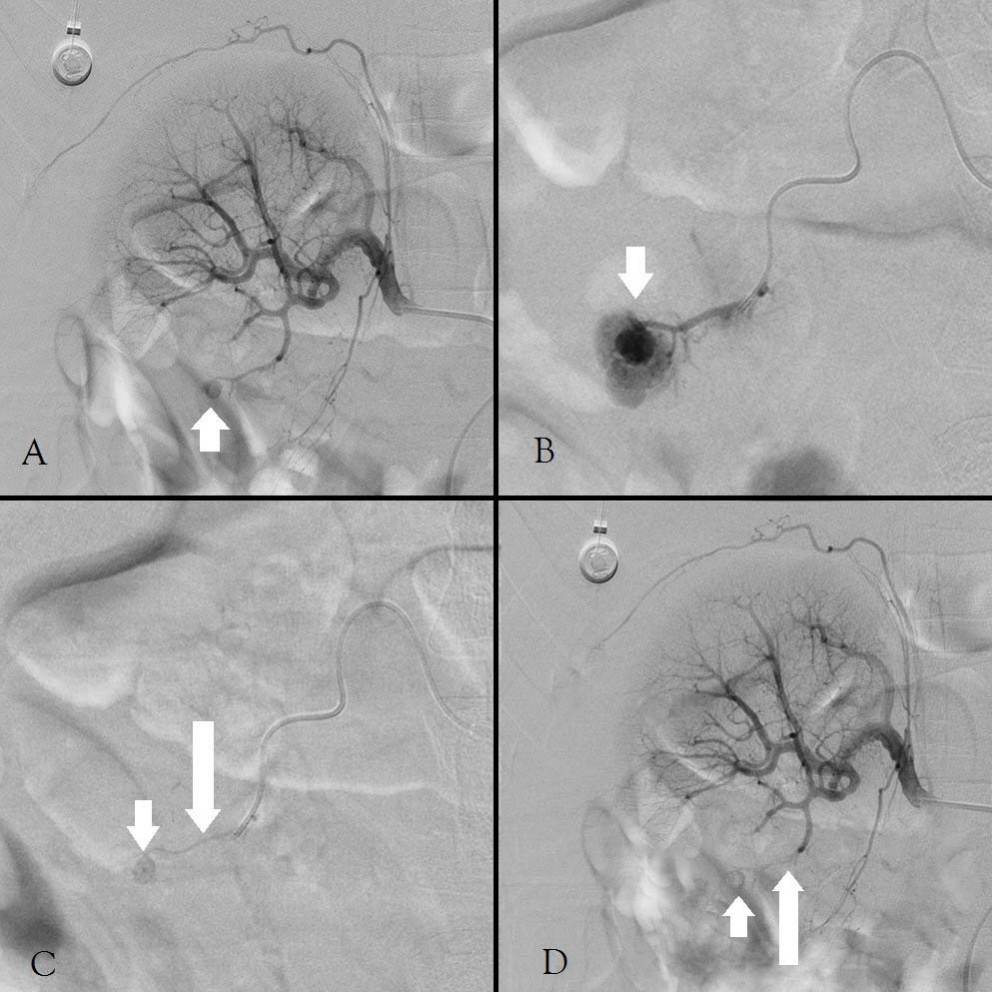
A 31-year-old woman presented with persistent hematuria after renal biopsy. (A) Arteriography of the right renal artery reveals a pseudoaneurysm (arrow) in a lower pole segmental artery. (B) A microcatheter was coaxially advanced through a 5-Fr Yashiro catheter, with the tip of the catheter situated a few millimeters proximal to the pseudoaneurysm. A test injection was performed which showed no reflux (arrow). (C) The FAL mixture was injected under fluoroscopic control, and the pseudoaneurysm (short arrow) and feeding artery were gradually filled with the glue mixture (long arrow). (D) The postembolization arteriogram confirms successful embolization of the target artery with complete resolution of the pseudoaneurysm (short arrow) and with preservation of the residual renal tissue (long arrow).

**Figure 2:**
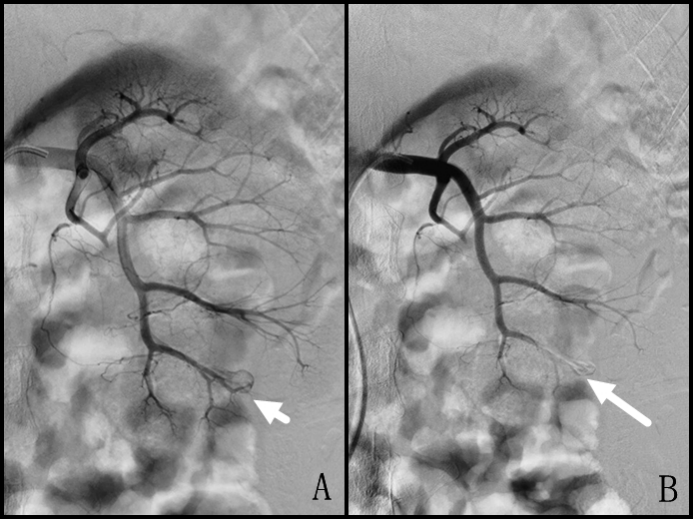
A 24-year-old man presented with persistent hematuria and hemorrhagic shock after percutaneous lithotripsy of renal stones. (A) Arteriography of the left renal artery reveals active contrast extravasation (short arrow) in a lower pole artery. (B) The postembolization arteriogram confirms successful embolization of the target artery (long arrow).

Technical success was achieved in all 14 cases with a single injection of glue (Fig. 1 and Fig. 2). Occlusion was observed immediately after the completion of FAL injection in all cases; no patients required a second FAL injection or the use of other embolic materials. The average procedure time was 15 minutes (range, 11–22 min). The mean injecting volume of FAL mixture was 0.5 mL (range, 0.2–0.8 mL) (Table 1). There was no reflux of glue outside the target vessel. The average cost of FAL for each procedure was $74 USD (range, $65–$130 USD; glue was $65 per vial and supplied in 0.5-mL aliquots). There were no complications related to the procedures.

Clinical success was achieved in all patients. Postprocedure transient hematuria occurred in 9 patients but resolved within 1 to 3 days in all cases. There was no recurrent hematuria or progression of hematoma identified in any patients, and no one required subsequent further endovascular or surgical intervention. There were no occurrences of fever, flank pain, leukocytosis, or any other signs or symptoms to suggest postembolization syndrome. Serum creatinine was normal in all cases with a mean follow-up of 4.3 months (range, 0.3–9.8 mo). There was no postprocedure hypertension in any patient. Postprocedure ultrasound and color Doppler examinations were obtained in all but 1 patient, with a mean follow-up interval of 15.1 months (range, 0.3–36.8 mo). There was no global kidney atrophy, parenchyma infarction, perirenal renal fluid collection, or renal abscess. All corresponding main and segmental renal arteries were patent.

## Discussion

This study demonstrated that a single FAL injection can be used safely and effectively to control significant peripheral renal arterial bleeding. When used with the coaxial technique and a microcatheter, FAL injection permits precise localization and catheterization of the bleeding arterial branches. Compared with partial or total nephrectomy, coaxial embolization reduces tissue loss because the embolization material can be deployed immediately proximal to the bleeding site. In this series, there was no nontarget embolization.

Transcatheter embolization of renal vascular lesions is currently the most effective treatment option for these patients, since this method definitively eliminates vascular lesions while preserving functional renal parenchyma^10^. With few exceptions, such as avulsion of the renal pelvis, injuries to the vascular pedicle, and life-threatening hemodynamic instability^14^, surgical intervention has become uncommon for the treatment of renal injuries as the availability of minimally invasive techniques has increased^15^.

The choice of embolic material is usually dependent on underlying pathology, vascular anatomy, availability of the embolic agent, and personal preference of the operator. Coils, the most commonly used embolic material, are typically employed for the occlusion of larger vessels. Coils can lead to complete occlusion equivalent to that seen with surgical ligation. With the use of a microcatheter, selective peripheral renal artery embolization can be achieved with minimal renal parenchyma loss^6^. However, coil embolization depends on the ability of the patient to form thrombus; coagulopathic states such as thrombocytopenia, platelet dysfunction, and abnormal clotting factors may hinder complete vessel occlusion. Furthermore, time to occlusion also depends on the type of coil used and the rate of flow of the target vessel embolized^16^. Choosing the proper type and size of coil can be challenging, as can precisely deploying the coil. Potential complications of coil embolization include occlusion of nontarget vessels and coil migration. The use of multiple coils may significantly increase the cost of the procedure^17^.

Gelfoam is a low-cost embolic agent that can be used for the treatment of bleeding peripheral renal arteries. However, in the renal setting, control of the distribution of Gelfoam can be difficult. In a study of iatrogenic and traumatic arteriovenous malformations treated with a conventional catheter technique (0.035″ system), Fisher et al^1^ found that up to 20% of the cases had renal function loss ranging from 30% to 50% because of nontarget embolization. If a microcatheter technique is used instead, large Gelfoam particles cannot be delivered to the area, so vascular occlusion may not be possible for a pseudoaneurysm or an actively bleeding vessel. Furthermore, the fine Gelfoam slurry can increase the risk of reflux to the nontarget vessel^18^. In renal artery pseudoaneurysm embolization, absorbable Gelfoam embolization increases the risk of rupture by increasing the pressure inside the cavity^19^,^20^. Therefore, many operators have chosen to abandon absorbable Gelfoam for this purpose.

The use of glue for the treatment of gastrointestinal bleeding was first reported in 1975^21^; since then, this agent has mostly been used secondary to coils and other materials^6^. Kish et al^8^ reported that using N-butyl cyanoacrylate (n-BCA) glue to treat recurrent renal vascular bleeding after failed microcoil or Gelfoam techniques had a 100% success rate in 10 patients. Recent studies have shown that liquid embolic material can be used as a first-line embolic agent for bleeding gastrointestinal vessels with a lower rate of recurrence^22^,^23^. In a study of n-BCA used for the embolization of renal arteries to treat a variety of underlying pathologies in 14 patients, technical success was achieved in all patients, with n-BCA used exclusively in 9 (64%) patients and in combination with an additional embolic material in 5 (36%) patients (4 coils, 1 Amplatzer Vascular Plug)^10^.

FAL is a medical glue made in China that contains high-purity (>99%) N-octyl cyanoacrylate (n-OCA) and n-BCA, which is mixed at the ratio of 1:4^24^. FAL has a polymerization time of 2 to 6 seconds with low polymerization enthalpy (1.6°C) and toughness in human tissue^24^,^25^. Compared with Onyx, FAL can be injected via normal microcatheter and prepared in few minutes. Onyx must be injected by using the Onyx liquid embolic system and needs to be shaken by an oscillator before use. FAL contains 25% n-OCA, which results in a prolonged polymerization time and more resilience versus n-BCA only^24^. This liquid embolic agent can be uniformly delivered to the target vessel with resultant rapid vascular occlusion. As with n-BCA, Onyx, and other liquid embolic agents, nontarget vascular occlusion can occur with FAL because of its liquid nature. Therefore, familiarity with polymerization time is the key to successfully performing embolization with FAL. Operators can control the polymerization time by choosing different ratios of FAL and Lipiodol, delivery speeds, and quantities of agents used. A FAL:Lipiodol ratio of 1:1 is ideal for embolizing segmental or subsegmental renal arteries, with a more diluted FAL mixture recommended for peripheral branches. A test injection with contrast medium should be administered first to determine the correct amount of FAL mixture injection and the appropriate administration speed. The actual FAL injection should use less volume and have a slower injection speed than the test injection, as the viscosity of the FAL mixture is different from that of contrast.

The major advantage of FAL is its significantly lower price (equivalent to $65 per vial) compared with n-BCA ($485) and Onyx ($1129) in our regional market. A single microcoil (Tornado, Cook, Bloomingdale, Indiana) costs approximately $290 at our institution. In a series of 9 cases of renal artery injuries similar to those seen in our study, Liu et a1^26^ reported that at least 4 microcoils (maximum 12) were needed to achieve complete occlusion. Therefore, the cost of the coil procedure would be much higher than the cost of the FAL injection at our institution.

The limitations of this study include its retrospective nature and small number of cases. The operators who performed the procedures were required to have experience with liquid embolics. The success we observed with embolization of renal arterial lesions may not necessarily apply to other visceral artery embolizations, as the renal artery does not have dual or multiple suppliers.

In conclusion, embolization with the liquid embolic agent FAL is effective for the treatment of active bleeding from peripheral renal arteries. This procedure has a high success rate and takes less time and is less expensive than embolization with other embolic agents.

## Author Contributions

T.A. and S.S.Z. collected data and wrote the main manuscript text. S.S.Z. contributed to the work equally and should be regarded as co-first author. M.X. prepared all of the figures and table. S.Z. designed the study. W.W. adjusted the structure of the manuscript and edited the figures and table. All authors reviewed the manuscript.

## Figures and Tables

**Table 1:** Clinical characteristics of 14 patients undergoing FAL embolization of the renal artery

No.	Age (y)/sex	Underlying condition	Clinical presentation	CT finding	Pre-procedure HGB (g/L)	Follow-up (mo)	Angiographic findings	Localization	Injected volume of FAL mixture (mL)	Procedure time (min)
1	17/M	Trauma	LBP and hematuria	SCH	65	6.3	Active contrast extravasation	RR/Interlobar artery	0.6	17
2	31/F	Renal biopsy	Hematuria	SCH	80	13.0	Pseudoaneurysm	RR/Interlobar artery	0.7	10
3	25/M	Renal biopsy	LBP	SCH	70	34.4	Pseudoaneurysm	RR/Arcuate artery	0.5	16
4	24/M	PRL	Hematuria	SCH	38	6.5	Active contrast extravasation	LR/Interlobar artery	0.6	20
5	17/F	Trauma	LBP and hematuria	RPH	70	19.0	Pseudoaneurysm	RR/Arcuate artery	0.8	15
6	12/M	Trauma	LBP and hematuria	SCH	75	0.2	Active contrast extravasation	LR/Arcuate artery	0.3	11
7	23/F	Trauma	LBP and hematuria	RPH	70	13.0	Pseudoaneurysm	LR/Arcuate artery	0.2	13
8	29/M	Trauma	Hematuria	SCH	66	12.7	Pseudoaneurysm	RR/Interlobar artery	0.6	13
9	62/M	PRL	LBP and hematuria	SCH	65	34.0	Pseudoaneurysm	RR/Interlobar artery	0.4	14
10	16/M	Trauma	LBP and hematuria	RPH	62	36.8	Pseudoaneurysm	RR/Interlobar artery	0.5	18
11	40/M	PRL	Hematuria	SCH	52	15.0	Pseudoaneurysm	LR/Arcuate artery	0.6	15
12	48/F	Renal biopsy	LBP	SCH	62	5.2	Pseudoaneurysm	RR/Interlobar artery	0.5	13
13	41/M	Complication of cardiac catheterization	LBP	SCH	85	14.7	Pseudoaneurysm	RR/Interlobar artery	0.4	16
14	42/M	Renal biopsy	LBP and hematuria	SCH	58	0.3	Active contrast extravasation	LR/Interlobar artery	0.6	22

PRL = percutaneous lithotripsy of renal stones; LBP = lower back pain; SCH = subcapsular hematoma; RPH = retroperitoneal hematoma; RR = right renal; LR = left renal; HGB = haemoglobin.
